# Systematic review and meta-analysis of analgesic treatment options in patients with rheumatoid arthritis related pain

**DOI:** 10.1186/s42358-025-00484-1

**Published:** 2025-10-17

**Authors:** Caleb Lautenschlager, Ashton Stropes, Andrew Smith, Maureen E. Knell, Morgan Sperry

**Affiliations:** 1 Drug Information Center, Division of Pharmacy Practice and Administration, University of Missouri-Kansas City School of Pharmacy, 2464 Charlotte Street, Kansas City, MO 64108 USA; 2 Division of Pharmacy Practice and Administration, University of Missouri-Kansas City School of Pharmacy, 2464 Charlotte Street, Kansas City, MO 64108 USA

**Keywords:** Rheumatoid arthritis, Pain management, Analgesics, Systematic review, Meta-analysis, Chronic pain

## Abstract

**Background:**

Rheumatoid arthritis is a chronic autoimmune disorder characterized by joint destruction and inflammation. Persistent pain is a common symptom, often impacting quality of life, physical function, and mental health. Despite its prevalence, limited guidance exists for managing chronic rheumatoid arthritis-related pain. This systematic review and meta-analysis aimed to evaluate the effectiveness of various analgesic therapies for managing rheumatoid arthritis-related pain symptoms.

**Methods:**

A comprehensive search of PubMed, EMBASE, Cochrane, and ClinicalTrials.gov was conducted from database inception through November 7, 2024. Eligible studies included randomized controlled trials and prospective cohorts reporting pain outcomes in adult rheumatoid arthritis patients. Pain score data was stratified by scale (5-point, 10-point, 100-point) and analyzed using a random-effects model.

**Results:**

Twenty-six studies covering 52 treatment regimens were included. Etodolac demonstrated significant pain reduction on a 5-point scale (200 mg: 3.24; 95% CI: 2.86 to 3.63; *p* < 0.001; 300 mg: 3.35; 95% CI: 2.96 to 3.74; *p* = 0.00). Piroxicam also showed benefit (20 mg: 3.35; 95% CI: 2.91 to 3.78; *p* < 0.001). On the 10-point scale, only extracorporeal shock wave therapy significantly reduced pain (3.36; 95% CI: 2.25 to 4.48; *p* < 0.001). Celecoxib (Celbesta) (200 mg BID) was most effective in the 100-point subgroup (1.73; 95% CI: 1.32 to 2.15; *p* < 0.001). Heterogeneity was high (I² = 91.1%, 94.1%, 60.2%; all *p* < 0.05), and publication bias was present.

**Conclusion:**

Several therapies demonstrated substantial pain relief, but heterogeneity, publication bias, and varying study quality limit generalizability. High-quality, long-term trials with standardized pain assessments are needed to guide personalized rheumatoid arthritis pain management.

**Clinical trial number:**

PROSPERO CRD42024593971, 4 October 2024.

## Background

Rheumatoid arthritis (RA) is a chronic autoimmune disorder characterized by systemic inflammation, leading to pain, stiffness, and tenderness in the joints [1]. In 2019, an estimated 18.5 million cases of RA were identified globally, with approximately 1.5 million individuals affected in the United States [2, 3]. Among these patients, over 70% experience moderate to severe disease states [4]. Rheumatoid arthritis predominantly affects women, who are three times more likely to develop the disease than men, and its incidence rises from age 5 to 54 years old with most patients starting to develop the condition between the ages of 30 and 60 [5]. The disease is driven by autoimmune-mediated joint destruction, leading to synovial inflammation and hyperplasia, along with elevated antibody levels such as rheumatoid factor [2]. Without appropriate treatment, RA progressively worsens, potentially causing irreversible joint and tissue damage. While current treatments cannot cure RA, they focus on symptom management, improving quality of life, and minimizing disease progression [1]. This is especially important as RA symptoms affect a younger demographic than other arthritis conditions. Consequently, optimizing symptom control, particularly stiffness and pain, is critical to preserving functional capacity and maintaining quality of life during the prime years of adulthood.

Patients with rheumatoid arthritis may experience periods of disease activity that fluctuate between active and inactive states. During inactive phases, symptoms are typically absent, while active disease presents with joint inflammation and pain. RA symptoms commonly affect smaller joints, such as those in the hands, wrists, and feet, but can also involve larger joints, including the knees, elbows, and shoulders [1].

The impact of RA extends beyond joint pain, significantly impairing quality of life, physical function, and mental health. As a systemic disease, RA can lead to a range of extra-articular manifestations, including fevers, vasculitis, neuropathy, pulmonary and cardiovascular disease, and the development of rheumatoid nodules in the lungs and other organs, all of which contribute to the overall disease and pain burden. Everyday activities such as writing, typing, and walking can become painful, making it difficult to complete routine tasks. Additionally, persistent pain often leads to poor sleep quality, loss of appetite, fatigue, and malaise [4]. Mental health complications are also prevalent, with RA patients experiencing higher rates of depression. Major depressive disorder (MDD) affects approximately 13–42% of RA patients, representing a two to four-fold increased risk compared to the general population [6].

According to the 2021 rheumatoid arthritis (RA) guidelines from the American College of Rheumatology, treatment strategies emphasize a “treat-to-target” approach which prioritizes early diagnosis, prompt initiation of disease-modifying anti rheumatic drugs (DMARDs) such as methotrexate (MTX), hydroxychloroquine, or sulfasalazine, and rigorous monitoring of disease activity. The therapeutic goal is to achieve remission or low disease activity through systematic treatment adjustments. While early and effective treatment with DMARDs can reduce inflammation and slow disease progression, by the time this is attained, irreversible joint damage or persistent inflammation may have already occurred, leading to chronic pain and impaired range of motion and function despite low disease activity [7].

Agents such as DMARDs primarily aim to reduce disease activity and slow disease progression, which may indirectly alleviate pain; however, DMARDs are not intended for direct analgesic purposes. Therefore, when additional analgesia is required, nonsteroidal anti-inflammatory drugs (NSAIDs) are the preferred option [8]. However, limited guidance exists on how to manage persistent pain in patients who do not respond adequately to NSAIDs. A general therapeutic objective for chronic pain management includes achieving a 30% reduction in pain severity. Corticosteroid injections provide temporary relief but are unsuitable for long-term use, leading many patients to rely on opioids [9]. Studies estimate that up to 40% of RA patients are consistent opioid users, despite concerns over reduced efficacy, dependence, and safety risks associated with prolonged use [10].

Systematic comparisons of analgesic efficacy in RA pain management are often limited to intra-class evaluations or focus exclusively on either pharmaceutical or non-pharmaceutical treatments. Given the lack of comprehensive guidelines for pain management and the growing concern over long-term opioid use, there is a critical need for a broader assessment of effective and alternative pain management strategies.

The present systematic review aims to evaluate the effectiveness of various analgesic therapies for managing RA-related pain symptoms.

## Methods

The statistical software used to synthesize collected data was IBM SPSS Statistics 29.0.1.1. Standard deviation of pain score results was used as the effect size measure. Additionally, a random-effects model was applied, accounting for potential variability between study effects. Heterogeneity was assessed using the I2 statistic. I2 values above 70% indicate considerable heterogeneity that may influence the validity of the results. Risk of bias was assessed using the appropriate Cochrane Risk of Bias Tool based on study design. Randomized trials were evaluated using the Risk of Bias 2 (RoB 2) tool, while non-randomized trials were assessed using the Risk of Bias in Non-Randomized Studies, Version 2 (ROBINS-I V2). Bias assessment was performed independently by two reviewers, with a third reviewer resolving any disagreements. Publication bias was assessed via an Egger’s test.

A systematic search was conducted through various databases including PubMed, EMBASE, the Cochrane Library, and ClinicalTrials.gov. Searches encompassed all available literature from database inception through November 7, 2024. Keywords utilized include rheumatoid arthritis, RA, analgesics, pain relievers, NSAIDs OR Nonsteroidal Anti-Inflammatory Drugs, opioids, acetaminophen, paracetamol, cox-2 inhibitors, corticosteroid, biologics, physical therapy, neural training, functional training, occupational therapy, ergotherapy, cognitive behavioral therapy, hypnotherapy, ultrasound therapy, acupuncture, thermotherapy, laser therapy, electrotherapy, balneotherapy, non-pharmaceutical, pain relief, pain reduction, pain intensity. Filters were applied, restricting results to studies involving human subjects and results were limited to randomized controlled trials, systematic reviews, meta-analyses, prospective cohorts, retrospective cohorts, and case-controlled studies. Complete search queries for each database can be found in Table 1.

**Table 1:** Search queries

Database	Search Query
EMBASE	‘rheumatoid arthritis’/exp/mj AND (‘comparative study’/exp OR ‘therapy’/exp OR ‘therapy’ OR ‘comparative effectiveness’/exp OR ‘comparative effectiveness’) AND (‘analgesia’/exp/mj OR ‘pain reduction’/exp/mj OR ‘pain intensity’/exp/mj) AND (‘analgesic agent’/exp OR ‘nonsteroid antiinflammatory agent’/exp OR ‘opiate’/exp OR ‘paracetamol’/exp OR ‘cyclooxygenase 2 inhibitor’/exp OR ‘corticosteroid’/exp OR ‘biological product’/exp OR ‘physiotherapy’/exp OR ‘physiotherapy’ OR ‘functional training’/exp OR ‘functional training’ OR ‘occupational therapy’/exp OR ‘occupational therapy’ OR ‘cognitive behavioral therapy’/exp OR ‘cognitive behavioral therapy’ OR ‘hypnotherapy’/exp OR ‘hypnotherapy’ OR ‘ultrasound therapy’/exp OR ‘ultrasound therapy’ OR ‘acupuncture’/exp OR ‘acupuncture’ OR ‘thermotherapy’/exp OR ‘thermotherapy’ OR ‘laser therapy’/exp OR ‘laser therapy’ OR ‘electrotherapy’/exp OR ‘electrotherapy’ OR ‘balneotherapy’/exp OR ‘balneotherapy’ OR ‘non pharmaceutical intervention’/exp OR ‘non pharmaceutical intervention’)
PubMed	(((rheumatoid arthritis[Title/Abstract])) AND ((comparative study) OR (comparative effectiveness) OR (compared to)) AND ((((pain management[Title/Abstract]) OR(pain relief[Title/Abstract])) OR (Pain reduction[Title/Abstract])) OR (Pain intensity[Title/Abstract]) AND ((medline[Filter]) AND (meta-analysis[Filter] OR randomizedcontrolledtrial[Filter] OR systematicreview[Filter]) AND (humans[Filter]) AND (english[Filter]))) AND ((Analgesics) OR (pain relievers) OR (NSAID) OR (opioids) OR (opiate) OR (acetaminophen) OR (paracetamol) OR (cox-2 inhibitors) OR (corticosteroids) OR (biologics) OR (physical therapy) OR (neural training) OR (functional training) OR (occupational therapy) OR (ergotherapy) OR (cognitive behavioral therapy) OR (hypnotherapy) OR (ultrasound therapy) OR (acupuncture) OR (thermotherapy) OR (laser therapy) OR (electrotherapy) OR (balneotherapy) OR (non-pharmaceutical)) AND ((meta-analysis[Filter] OR randomizedcontrolledtrial[Filter] OR systematicreview[Filter]) AND (humans[Filter]) AND (english[Filter])))
Clinicaltrials.gov	Rheumatoid Arthritis | Other terms: Pain Management | Analgesics OR Pain relievers OR NSAIDs OR Nonsteroidal Anti-Inflammatory Drugs OR Opioids OR Acetaminophen OR Paracetamol OR Cox-2 inhibitors OR Corticosteroids OR Biologics OR Physical therapy OR Neural training OR Functional training OR Occupational therapy OR Ergotherapy OR Cognitive behavioral therapy OR Hypnotherapy OR Ultrasound therapy OR Acupuncture OR Thermotherapy OR Laser therapy OR Electrotherapy OR Balneotherapy OR Non-pharmaceutical | Adult (18–64), Older adult (65+) | Studies with results | Study completion on or before 11/07/2024
Cochrane Library	(“rheumatoid arthritis”):ti, ab, kw AND ((“comparison studies”):ti, ab, kw OR (“comparison study”):ti, ab, kw OR (“efficacy studies”):ti, ab, kw (Word variations have been searched)) AND ((“pain management”):ti, ab, kw OR (“pain relief”):ti, ab, kw OR (“pain reduction”):ti, ab, kw OR (“pain intensity”):ti, ab, kw OR (analgesic): ti, ab, kw OR (“non-steroidal anti-inflammatory agent”):ti, ab, kw OR (opioids): ti, ab, kw OR (“acetaminophen”):ti, ab, kw OR (paracetamol): ti, ab, kw OR (“paracetamol”):ti, ab, kw OR (“COX-2 inhibitor”):ti, ab, kw OR (“corticosteroid”):ti, ab, kw OR (“biologic”):ti, ab, kw OR (“physical therapy”):ti, ab, kw OR (neural training): ti, ab, kw OR (functional training): ti, ab, kw OR (occupational training): ti, ab, kw OR (ergotherapy): ti, ab, kw OR (“cognitive behavior therapy”):ti, ab, kw OR (hypnotherapy): ti, ab, kw OR (ultrasound therapy): ti, ab, kw OR (“acupuncture”):ti, ab, kw OR (“thermotherapy”):ti, ab, kw OR (“laser therapy”):ti, ab, kw OR “electrotherapy” in Title Abstract Keyword OR “balneotherapy” in Title Abstract Keyword OR “non-pharmaceutical” in Title Abstract Keyword)

Zotero (v7.0.11) was used to manage references and remove duplicate records across databases. Two independent reviewers screened titles and abstracts to exclude irrelevant studies. Any disagreements were resolved by a third independent reviewer. Studies were included if they examined adult patients (≥ 18 years) with RA experiencing disease-related pain and reported pain-related outcomes following analgesic treatment. Studies were excluded if they were non-comparative, involved non-human subjects, or included a mixed disease population without a clear distinction between groups in the results. Additionally, trials were excluded if pain outcomes could not be clearly attributed to active RA, without other potential comorbidities affecting pain relief. Although it is challenging to ensure that pain outcomes solely reflect disease related effects, these exclusions provide sufficient confidence that the outcomes analyzed accurately reflect RA-related pain relief. A comprehensive list of inclusion and exclusion criteria is detailed in Table 2.

**Table 2:** Inclusion and exclusion criteria

Category	Inclusion criteria	Exclusion criteria
Population	Adult patients (18+) diagnosed with rheumatoid arthritis experiencing disease-related pain. Human studies only.	Patients with non-rheumatoid arthritis related pain. Studies with mixed patient population where data specific to rheumatoid arthritis is not clearly distinguished.
Intervention	Studies assessing the effectiveness of various analgesic treatments including both pharmaceutical and non-pharmaceutical therapies. Pharmaceutical therapies include NSAIDs, opioids, acetaminophen, paracetamol, cox-2 inhibitors, biologics, and corticosteroids. Non-pharmaceutical therapies include physical therapy, neural training, functional training, occupational therapy, ergotherapy, cognitive behavioral therapy, hypnotherapy, ultrasound therapy, acupuncture, thermotherapy, laser therapy, electrotherapy, and balneotherapy.	Studies focused on treatments unrelated to pain management or not utilizing analgesic therapies including DMARDs that do not primarily target pain.
Comparator	Studies comparing various analgesic treatment or interventions including drug versus drug, drug versus non-pharmaceutical intervention, drug versus placebo, non-pharmaceutical versus non-pharmaceutical, non-pharmaceutical versus placebo.	Studies without a comparison between different treatments or interventions
Outcomes	Outcomes must be related to pain relief, pain management, pain severity scores reduction of pain intensity or maintenance of pain symptoms.	Studies that do not report pain-related outcomes
Timing	All study durations and follow ups will be included.	None
Study Design	RCT’s, cohort studies (prospective and retrospective), and case-controlled studies.	Case series, case reports, editorials, opinion pieces, letters, animal studies or in vitro studies
Publication Language and Dates	No limits to publication date. English language abstracts of non-English articles will be reviewed alongside English written articles.	Abstracts without a published study manuscript

NSAIDs = nonsteroidal anti-inflammatory drugs, RCT = randomized controlled trial, DMARD = disease-modifying antirheumatic drugs

Eligible studies underwent data extraction. Relevant data collected included treatment regimen, randomization method, pain scale utilized (5-point, 10-point, or 100-point scale), number of participants in the treatment group, mean pain scores at baseline and post-treatment, mean change in pain score, associated standard deviations, 95% confidence intervals (CIs), and p-values. Data extraction was conducted independently by two reviewers and subsequently compared for consistency.

**Fig. 1:**
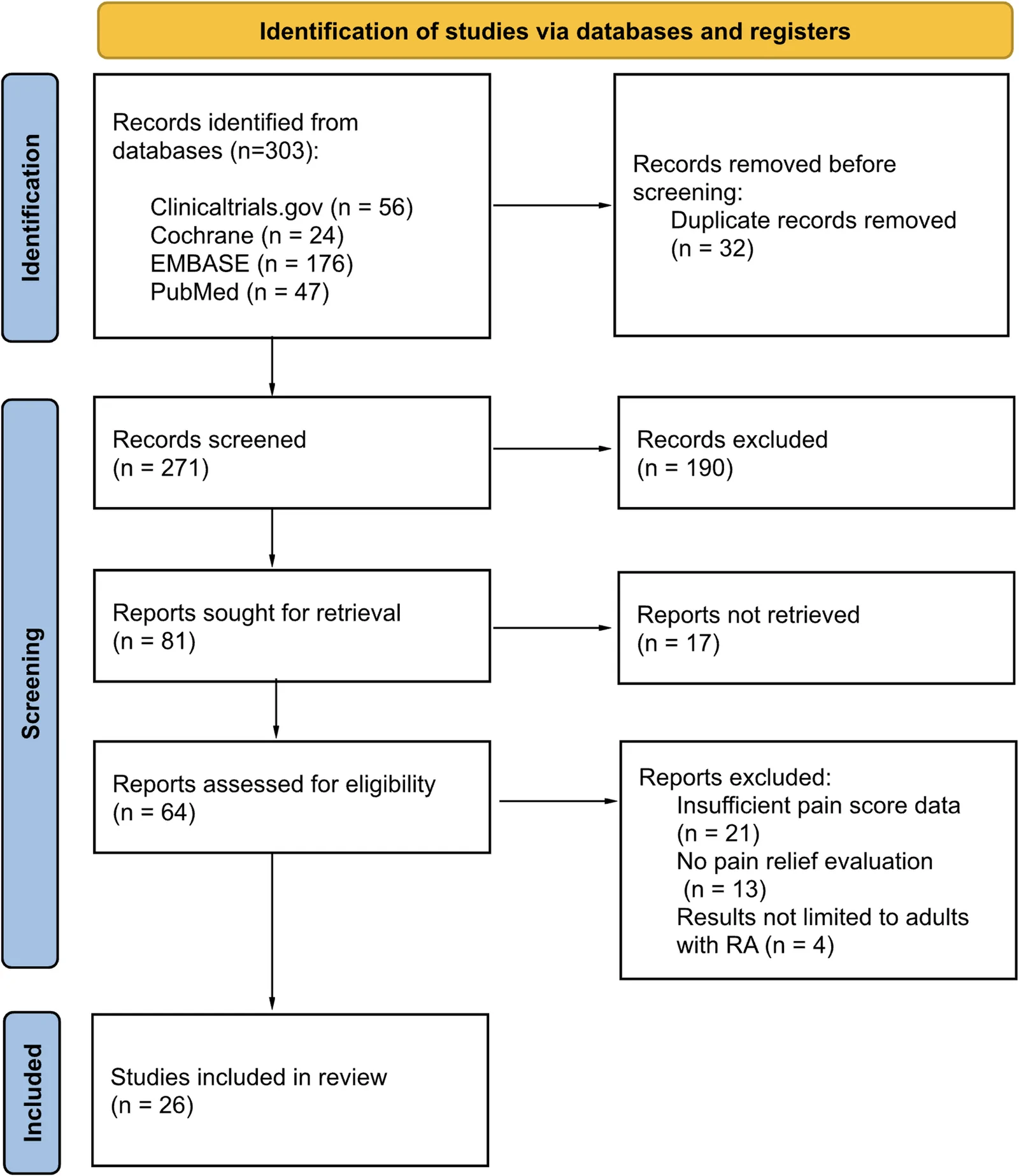
Identification of studies

The results of the literature search are summarized in Fig. 1. A total of four databases were searched, yielding 303 articles. After removing duplicate records, 271 articles remained for title and abstract screening. Based on the pre-established inclusion and exclusion criteria, 81 articles were deemed eligible for full-text review; however, 17 of these texts could not be retrieved. Following further evaluation of the remaining eligible articles, 38 studies were excluded for the following reasons: 21 articles provided insufficient pain score data, 13 articles did not evaluate pain relief, and 4 articles did not focus exclusively on adults with RA-related pain. Ultimately, 26 studies met the inclusion criteria and were utilized in the final systematic analysis.

Studies included ranged from randomized controlled trials, crossover trials, and prospective case series. Sample sizes varied from 15 to over 700 participants, with most studies utilizing a 100-point visual analog scale (VAS) to measure pain outcomes. Interventions ranged from NSAIDs, opioids, biologics, and various novel non-pharmaceutical options. A single trial observed the difference of celecoxib (Celebrex) brand name to celecoxib (Celbesta, an international generic equivalent) [11]. Due to the trials intention and generic comparator, the brand name Celebrex and Celbesta will be used throughout the review to provide clarity in results. Refer to Table 3 for more details regarding study characteristics.

**Table 3:** Study characteristics

Author (publication year)	Study design	Number of participants	Treatment regimen(s)	Pain scale utilized
Ash, G., et al. (1999) [26]	double-blind, placebo-controlled, randomized	25	dothiepin	100-point VAS
Bernateck, Michael, et al. (2008) [18]	randomized controlled trial	44	autogenic training, electroacupuncture	10-point VAS
Bickham, Kara, et al. (2016) [27]	double-blind, randomized controlled trial	713	etoricoxib	100-point VAS
Chalmers, A. C., et al. (2000) [19]	randomized, crossover trial	24	soft orthoses shoe supports, semi-rigid orthoses shoe supports	10-point VAS
Chang, C.-K., et al. (2021) [28]	randomized, prospective trial	36	TCZ, JAKi	100-point VAS
Choi, In Ah, et al. (2014) [29]	multicenter, randomized, double-blind, non-inferiority trial	120	celecoxib, pelubiprofen	100-point VAS
de Queiros, M. F. (1991) [14]	randomized, double-blind trial	39	etodolac, naproxen	5-point VAS
Emery, P., et al. (1992) [32]	multicenter, double-blind, randomized, parallel group trial	301	naproxen, nabumetone, nefopam	100-point VAS
Grace, E.M., et al. (1985) [15]	controlled, double-blind, randomized trial	18	amitriptyline	5-point VAS
Greenwald, M., et al. (2011) [31]	randomized, double-blind, placebo-controlled trial	600	etoricoxib	100-point VAS
Huskisson, E. C. (1991) [22]	single-blind, crossover trial	94	naproxen, diclofenac, indomethacin, piroxicam	10-point VAS
Karagülle, Mine, et al. (2017) [23]	single-blind, randomized controlled trial	37	DMARD (methotrexate, hydroxychloroquine, leflunomide, or sulfasalazine) + NSAID, spa therapy (saline balneotherapy)	100-point VAS
Kawai, Shinichi, et al. (2010) [32]	randomized, double-blind, placebo-controlled trial	338	ketoprofen	100-point VAS
Keystone, E., et al. (2008) [33]	randomized, placebo-controlled trial	298	rituximab + MTX	100-point VAS
Kim, Hyun-Sook, et al. (2020) [11]	multicenter, double-blind, double-dummy, active-controlled, randomized, parallel-group, non-inferiority trial	119	celecoxib (Celebrex), celecoxib (Celbesta)	100-point VAS
Lee, Yvonne C., et al. (2016) [21]	randomized, double-blind, crossover trial	23	milnacipran	10-point brief pain inventory
Leibing, E., et al. (1999) [34]	prospective, single-blinded, randomized control trial	19	cognitive-behavioral therapy	100-point VAS
Lightfoot, R (1997) [12]	randomized, double-blind, parallel group trial	338	etodolac, piroxicam	5-point VAS
Liu, Y., et al. (2018) [20]	prospective, case series trial	15	radial extracorporeal shock wave	10-point VAS
Mills, S. B., et al. (1973) [16]	randomized, double-blind, cross-over trial	34	ketoprofen, ibuprofen	5-point VAS
Preston, S. J., et al. (1989) [13]	randomized, cross-over trial	18	acetylsalicylic acid, sodium salicylate	5-point Likert scale
Raffaeli, William, et al. (2010) [24]	prospective, open-label, case series trial	24	oxycodone/acetaminophen	100-point VAS
Sánchez Andrada, S., et al. (1991) [25]	double-blind, randomized, control trial	20	indomethacin, droxicam	100-point VAS
Song, Yeong Wook, et al. (2007) [35]	multicenter, randomized, double-blind, double-dummy, non-interiority trial	174	celecoxib, SKI306X	100-point VAS
Waltham-Weeks, C. D., et al. (1987) [17]	randomized, double-blind, crossover trial	30	etodolac, naproxen	5-point VAS
Wang, Chien-Chih, et al. (2017) [36]	prospective, double-blind, randomized control trial	44	hyaluronic acid, lidocaine	100-point VAS

VAS = Visual analogue scale, TCZ = Tocilizumab, JAKi = Janus kinase inhibitor, DMARD = Disease modifying antirheumatic drugs, NSAID = Non-steroidal anti-inflammatory drugs, MTX = Methotrexate, SKI306X = Clematis mandshurisca, prunella vulgaris and trichosanthes kirilowii

**Fig. 2:**
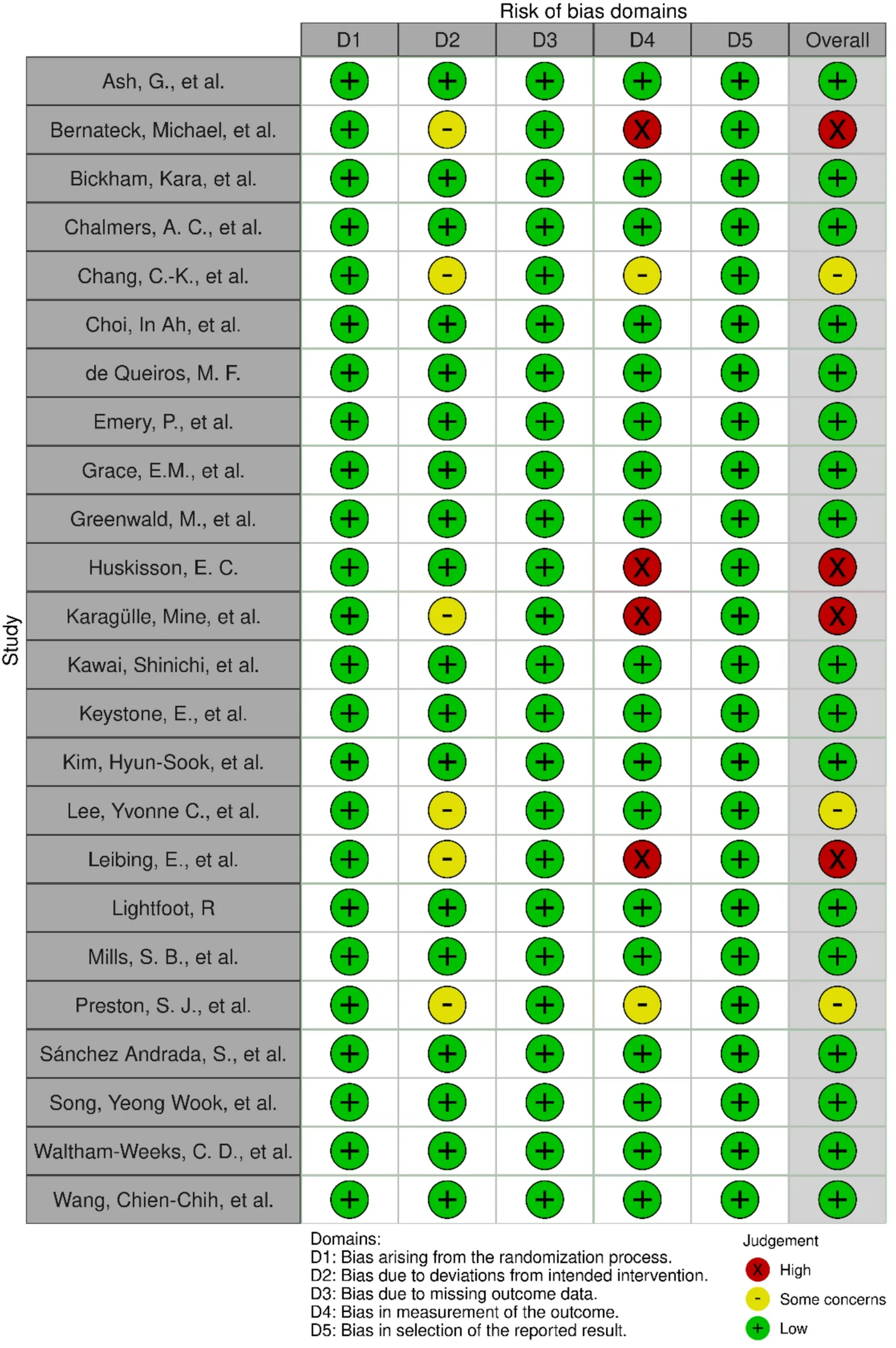
RoB 2 trial bias results

**Fig. 3:**
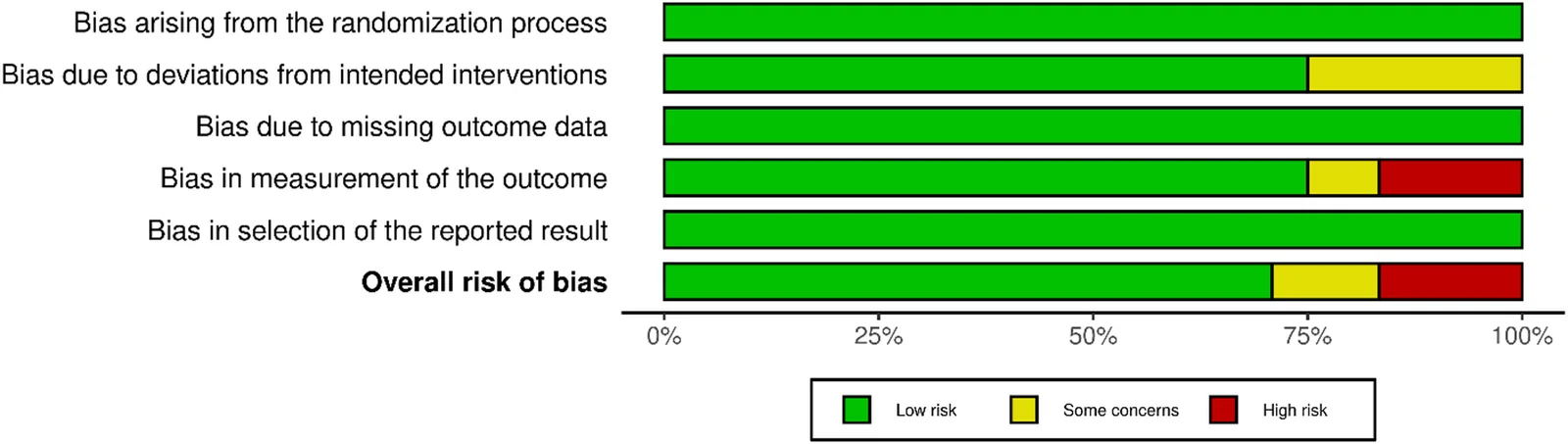
RoB 2 overall bias results

**Fig. 4:**
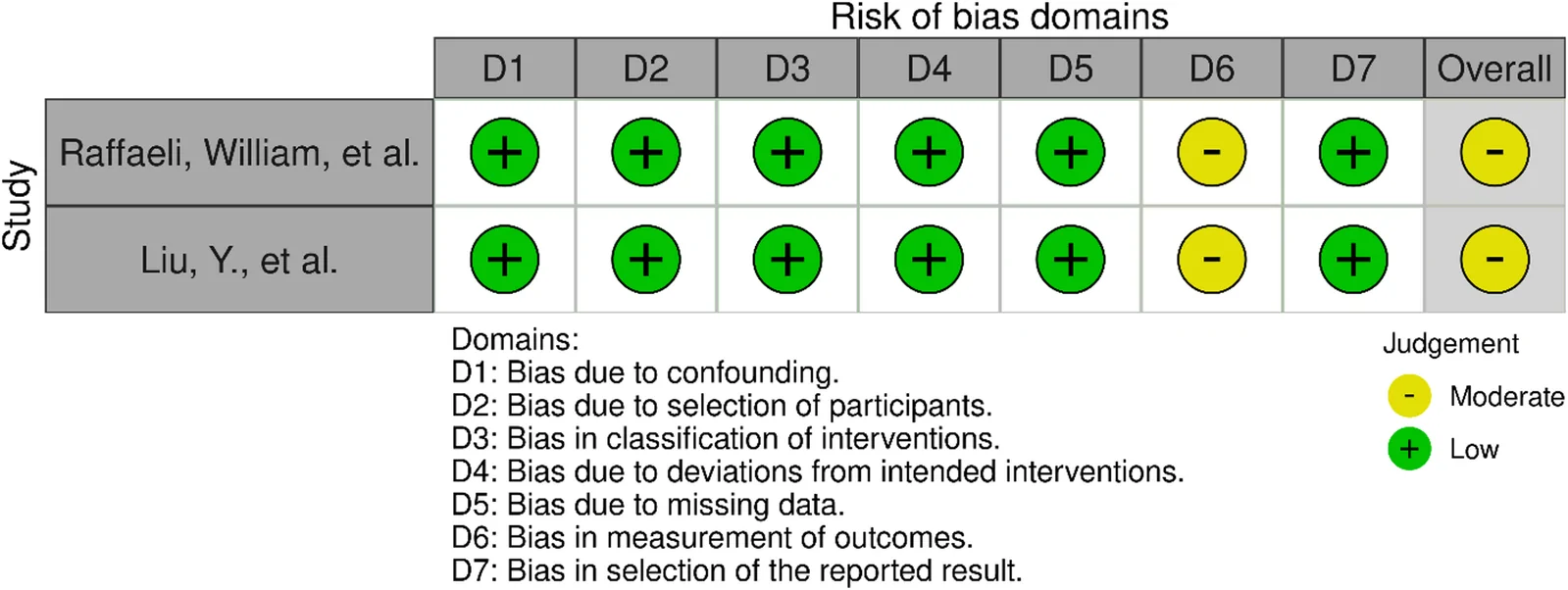
ROBINS-I V2 trial bias results

**Fig. 5:**
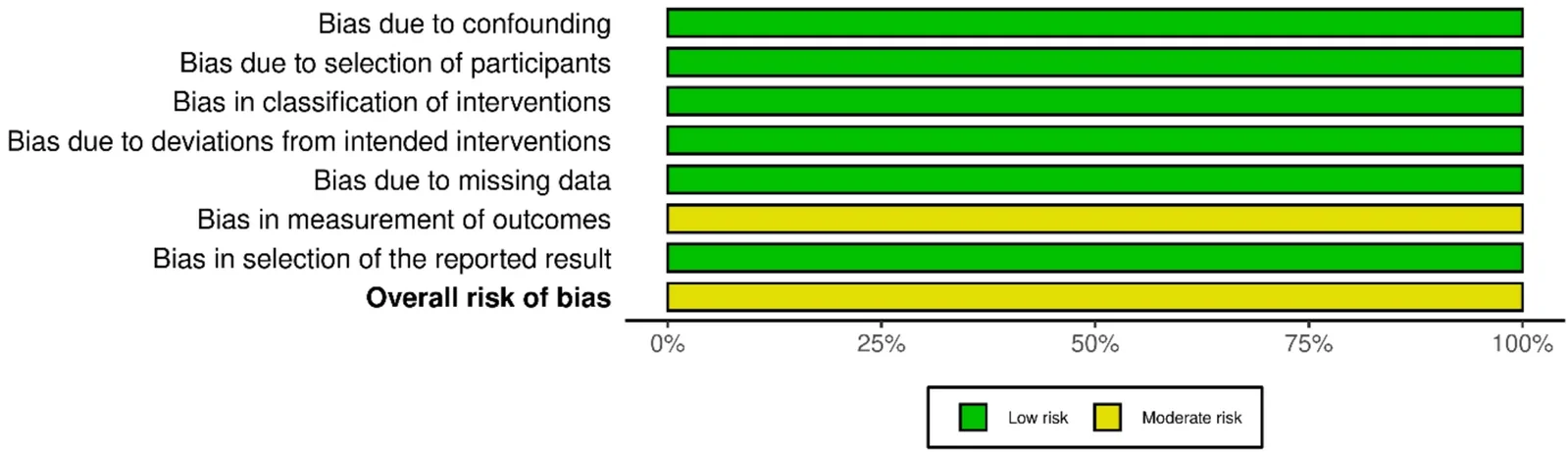
ROBINS-I V2 overall bias results

The risk of bias assessment utilized the RoB 2 tool for randomized trials while non-randomized trials were evaluated using the ROBINS-I-V2 tool. Overall, the assessment revealed that most studies presented with a low risk of bias among all domains (Figs. 2, 3, 4 and 5). Some studies however demonstrated concern largely around bias due to deviations from intended intervention and bias in measurement of outcomes. Both domain limitations often occurred due to a lack of blinding, either of the patient or the evaluator. These limitations are exacerbated by the pain score outcome being subjective in nature and susceptible to potential influence.

## Results

Analysis of pain data from the 26 included studies identified three distinct pain scales, which were used to create subgroups for the meta-analysis. These subgroups consisted of six trials utilizing a 5-point scale (5-point VAS or 5-point Likert scale), five trials using a 10-point scale (10-point VAS, 10-point brief pain inventory), and 15 trials employing a 100-point scale (100-point VAS). This stratification was implemented to account for differences in the granularity and precision of pain measurement, as well as potential variations in patient responses based on the scale presented to them. Overall, 52 treatment regimens from the included 26 studies were included in the systematic review (5-point scale: 14 regimens; 10-point scale: 10 regimens; 100-point scale: 28 regimens). Of those 52 regimens, 17 provided necessary results to conduct a meta-analysis (5-point scale: 5 regimens; 10-point scale: 5 regimens; 100-point scale: 7 regimens). Results for each treatment regimen in each subgroup can be found in Tables 4, 5 and 6.

**Table 4:** 5-Point scale results

Author	Treatment	Mean pain score at baseline (SD)	Mean pain score after treatment (SD)	Mean change in pain score after treatment (SD)
de Queiros, M. F. ^a^	etodolac (200 mg BID)	3.4	2.2	-1.2
de Queiros, M. F. ^a^	naproxen (500 mg BID)	3.3	2.3	-1
Grace, E.M., et al. ^a^	amitriptyline	2.44	1.5	-0.94
Lightfoot, R ^a^	etodolac (200 mg BID)	3.6 (0.7)	0.8 (1)	-2.8
Lightfoot, R ^a^	etodolac (300 mg BID)	3.7 (0.7)	1 (0.9)	-2.7
Lightfoot, R ^a^	piroxicam (20 mg)	3.6 (0.7)	0.9 (0.9)	-2.7
Mills, S. B., et al. ^a^	ketoprofen (50 mg TID)	2.39	2.28	-0.11
Mills, S. B., et al. ^a^	ketoprofen (50 mg TID)	2.37	2.05	-0.32
Mills, S. B., et al. ^a^	ibuprofen (400 mg TID)	2.39	2.45	0.06
Mills, S. B., et al. ^a^	ibuprofen (400 mg TID)	2.37	2.56	0.19
Preston, S. J., et al. ^b^	acetylsalicylic acid (4.8 g)	3.06 (0.54)	1.94 (0.8)	-1.12
Preston, S. J., et al. ^b^	sodium salicylate (4.8 g)	3.06 (0.54)	1.89 (0.76)	-1.17
Waltham-Weeks, C. D., et al. ^a^	etodolac (200 mg BID)	--	--	-1.1
Waltham-Weeks, C. D., et al. ^a^	naproxen (500 mg BID)	--	--	-0.73

a: Visual analog scale

b: 5-point Likert scale

**Table 5:** 10-Point scale results

Author	Treatment	Mean pain score at baseline (SD)	Mean pain score after treatment (SD)	Mean change in pain score after treatment (SD)
Bernateck, Michael, et al. ^a^	autogenic training	4.27 (1.37)	3.95 (2)	-0.32
Bernateck, Michael, et al. ^a^	electroacupuncture	4.6 (1.64)	2.79 (1.22)	-1.81
Chalmers, A. C., et al. ^a^	soft orthoses shoe supports	4 (2.37)	4.27 (0.45)	0.27
Chalmers, A. C., et al. ^a^	semi-rigid orthoses shoe supports	3.02 (2)	2.88 (0.44)	-0.14
Huskisson, E. C. ^a^	naproxen (1000 mg)	6.5	5.6	-0.9
Huskisson, E. C. ^a^	diclofenac (100 mg)	6.5	6	-0.5
Huskisson, E. C. ^a^	indomethacin (75 mg)	6.5	5.9	-0.6
Huskisson, E. C. ^a^	piroxicam (20 mg)	6.5	5.7	-0.8
Lee, Yvonne C., et al. ^b^	milnacipran (50 mg BID)	--	--	-0.67
Liu, Y., et al. ^a^	radial extracorporeal shock wave	5.7 (1.33)	1.33 (0.63)	-3.5

a: Visual analog scale

b: 10-point brief pain inventory

**Table 6:** 100-Point scale results

Author ^a^	Treatment	Mean pain score at baseline (SD)	Mean pain score after treatment (SD)	Mean change in pain score after treatment (SD)
Ash, G., et al.	dothiepin (150 mg)	56.7	42.2	-14.5
Bickham, Kara, et al.	etoricoxib (60 mg)	--	--	-30.96
Bickham, Kara, et al.	etoricoxib (90 mg)	--	--	-28.25
Chang, C.-K., et al.	TCZ	80	32.5	-52.5
Chang, C.-K., et al.	JAKi	87.5	24	-70
Choi, In Ah, et al.	celecoxib (200 mg BID)	64	42.7	-21.2 (20.8)
Choi, In Ah, et al.	pelubiprofen (30 mg TID)	69.1	42.9	-26.2 (19.5)
Emery, P., et al.	naproxen (1000 mg)	44.3 (23)	--	-2.3 (27)
Emery, P., et al.	nabumetone (2000 mg)	47.2 (22)	--	-6.3 (26)
Emery, P., et al.	nefopam (60 mg TID)	59	23	-36
Greenwald, M., et al.	etoricoxib (10 mg)	72.4 (17.3)	--	-21.42
Greenwald, M., et al.	etoricoxib (30 mg)	71 (16.7)	--	-23.11
Greenwald, M., et al.	etoricoxib (60 mg)	69.5 (18.2)	--	-23.01
Greenwald, M., et al.	etoricoxib (90 mg)	71.1 (17.6)	--	-31.23
Karagülle, Mine, et al.	DMARD (methotrexate, hydroxychloroquine, leflunomide, or sulfasalazine) + NSAID	60.45 (23.07)	50.27 (29.94)	-10.18
Karagülle, Mine, et al.	spa therapy (saline balneotherapy)	64.33 (25.62)	34 (19.77)	-30.33
Kawai, Shinichi, et al.	ketoprofen patch (100 mg)	50.1 (15.1)	--	-15.7 (16)
Keystone, E., et al.	rituximab + MTX	64.08 (22.28)	--	-23.37 (29.35)
Kim, Hyun-Sook, et al.	celecoxib (Celebrex) (200 mg BID)	55.7 (14)	35.5 (23.8)	-22.3 (3.1)
Kim, Hyun-Sook, et al.	celecoxib (Celbesta) (200 mg BID)	56 (13.4)	26.8 (19.7)	-31.3 (3.2)
Leibing, E., et al.	cognitive-behavioral therapy	41.8 (23.7)	34.5	-7.3
Raffaeli, William, et al.	oxycodone/ acetaminophen (5 mg/325 mg)	65.9 (15.8)	44.2 (24.7)	-21.7
Sánchez Andrada, S., et al.	indomethacin (100 mg)	57.7 (30.4)	29.7 (24.1)	-28
Sánchez Andrada, S., et al.	droxicam (20 mg)	49 (22)	17.1 (16.6)	-31.9
Song, Yeong Wook, et al.	celecoxib (200 mg BID)	--	--	-17.87 (19.06)
Song, Yeong Wook, et al.	SKI306X (200 mg TID)	--	--	-18.38 (20.8)
Wang, Chien-Chih, et al.	hyaluronic acid injection	53.3 (13.9)	--	-28.3 (11.1)
Wang, Chien-Chih, et al.	lidocaine injection	60.4 (17.56)	--	-17.3 (10.2)

a: Visual analog scale

TCZ = Tocilizumab, JAKi = Janus kinase inhibitor, DMARD = Disease modifying antirheumatic drugs, NSAID = Non-steroidal anti-inflammatory drugs, MTX = Methotrexate, SKI306X = Clematis mandshurisca, prunella vulgaris and trichosanthes kirilowii

Of the six trials utilizing a 5-point pain scale (Table 4), two provided sufficient data evaluation within the trial to complete further statistical analysis among a total of 356 patients [12, 13]. Four trials were excluded due to nonpositive variance or standard error [14–17]. Interventions included in the statistical analysis were etodolac 200 mg by mouth twice daily (118 patients), etodolac 300 mg by mouth twice daily (122 patients), piroxicam 20 mg by mouth daily (98 patients), acetylsalicylic acid 4.8 g by mouth daily (18 patients, crossover with sodium salicylate) and sodium salicylate 4.8 g by mouth daily (18 patients, crossover with acetylsalicylic acid).

**Fig. 6:**
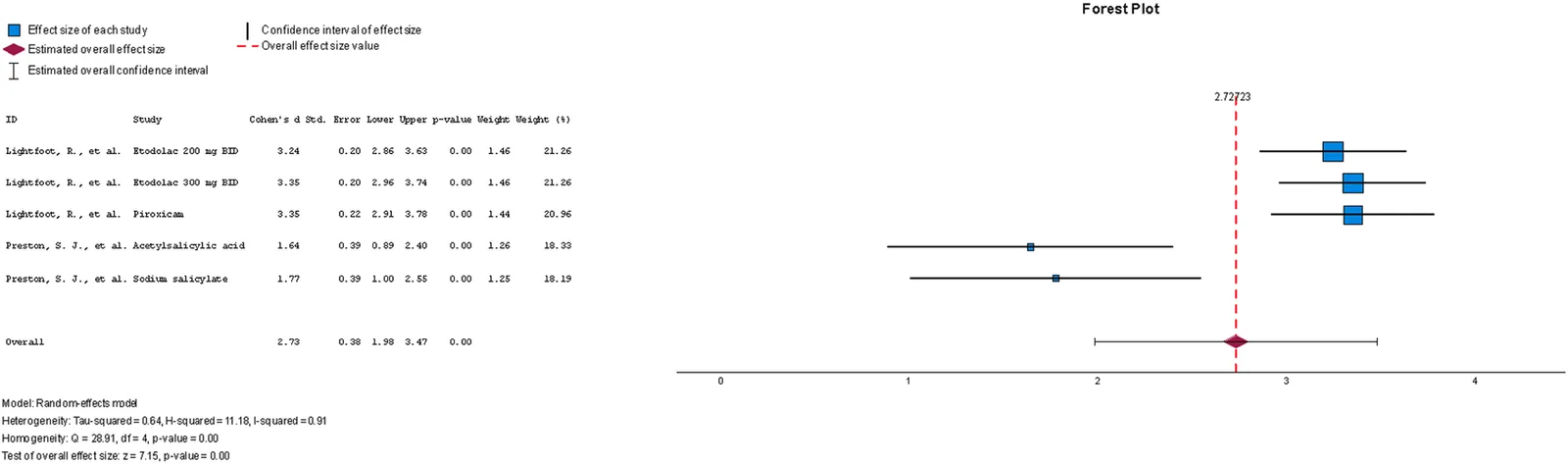
5-point pain scale meta-analysis results

The results of the meta-analysis indicated a significant decrease in pain scores among all five treatment options (*p* < 0.001). When viewed individually, both Etodolac doses (200 mg: 3.24, 95% CI: 2.86 to 3.63, *p* = 0.00; 300 mg: 3.35, 95% CI: 2.96 to 3.74, *p* = 0.00) as well as piroxicam (3.35, 95% CI: 2.91 to 3.78, *p* = 0.00) showed significantly improved pain relief over acetylsalicylic acid (1.64, 95% CI: 0.89 to 2.4, *p* = 0.00) and sodium salicylate (1.77, 95% CI: 1 to 2.55, *p* = 0.0) (Fig. 6).

The analysis of heterogeneity among the included studies revealed substantial variability in effect sizes, as indicated by the chi-square test (Q = 28.910, *p* < 0.001). The I² value of 91.1% also suggests a high degree of heterogeneity. These results led to the use of a random-effects model. Egger’s regression test for publication bias indicated significant asymmetry and the potential presence of publication bias or small-study effects (*p* = 0.001). Both heterogeneity and publication bias results are likely due to the nature of the meta-analysis question at hand and the lack of available clinical trials evaluating pain with a 5-point scale.

Of the five trials utilizing a 10-point pain scale (Table 5), three provided sufficient data to complete a statistical analysis among a total of 107 patients [18–20]. Two trials were excluded due to nonpositive variance or standard error [21, 22]. Interventions included in the statistical analysis were autogenic training (22 patients), a relaxation technique that can be practiced independently or incorporated as a component of cognitive behavioral therapy; electroacupuncture (22 patients), a variation of traditional acupuncture in which a low-intensity electrical current is applied through the acupuncture needles to enhance the therapeutic effect; soft orthoses shoe supports (24 patients); semi-rigid orthoses shoe supports (24 patients); and radial extracorporeal shock wave therapy (ESWT) (15 patients), the transcutaneous administration of high-energy acoustic waves to stimulate tissue healing and repair.

**Fig. 7:**
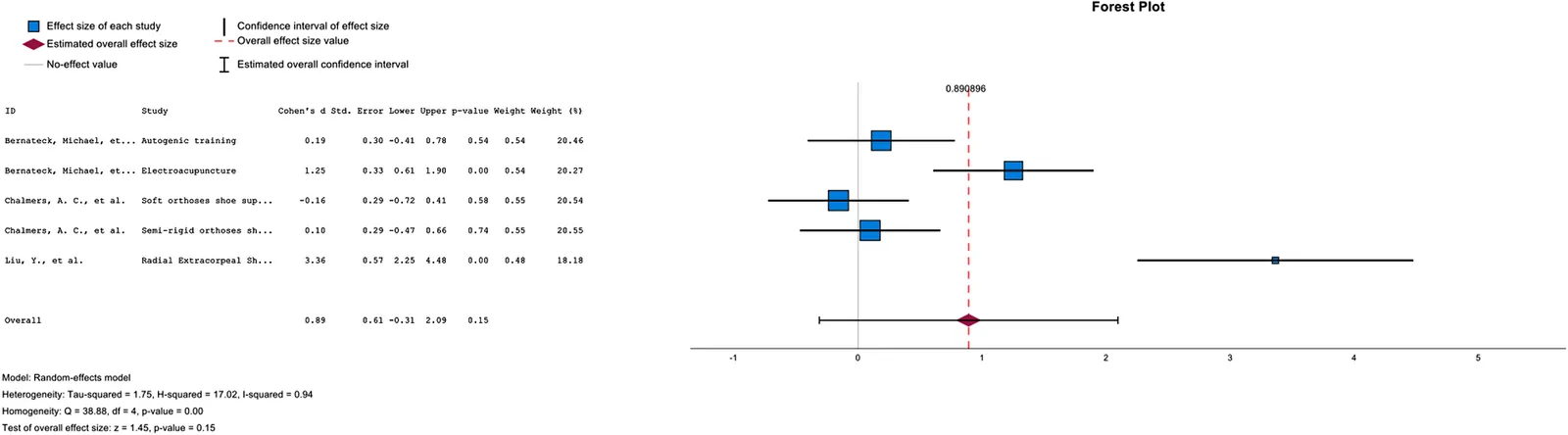
10-point pain scale meta-analysis results

The results indicated that autogenic training, soft orthoses, and semi-rigid orthoses did not significantly improve pain scores (*p* = 0.41, *p* = 0.66, *p* = 0.54, respectively). Meanwhile, while electroacupuncture improved overall pain scores, it did not demonstrate a significant advantage over other treatments (autogenic training, shoe supports and ESWT) in this group of studies (1.25, 95% CI: 0.61 to 1.9, *p* = 0.00). Among the five therapy options evaluated, only radial ESWT significantly reduced pain scores compared to other eligible interventions (3.36, 95% CI: 2.25 to 4.48, *p* = 0.00) (Fig. 7).

Heterogeneity analysis revealed similar results in the 10-point group as in the 5-point group, with substantial overall variability identified through the chi-square test (Q = 38.884, *p* < 0.001). Additionally, I² was 94.1%, necessitating the use of a random-effects model. Egger’s regression test indicated significant asymmetry and potential publication bias (*p* = 0.022). These findings are again likely attributable to the wide range of clinical trials included and the limited number of eligible trials within the 10-point scale subgroup.

Of the fifteen trials utilizing a 100-point pain scale (Table 6), four provided sufficient data to complete a statistical analysis among a total of 200 patients [11, 23–25]. The remaining eleven trials were excluded due to nonpositive variance or standard error [26–36]. Interventions that qualified for the statistical analysis includes celecoxib (Celebrex) 200 mg by mouth twice daily (58 patients), celecoxib (Celbesta) 200 mg by mouth twice daily (61 patients), DMARD (methotrexate, hydroxychloroquine, leflunomide, or sulfasalazine) + NSAID (22 patients), spa therapy with saline balneotherapy (15 patients), a practice where patients bathe in salt water for medicinal purposes, oxycodone 5 mg/acetaminophen 325 mg by mouth daily (24 patients), indomethacin 100 mg by mouth daily (10 patients), and droxicam 20 mg by mouth daily (10 patients).

**Fig. 8:**
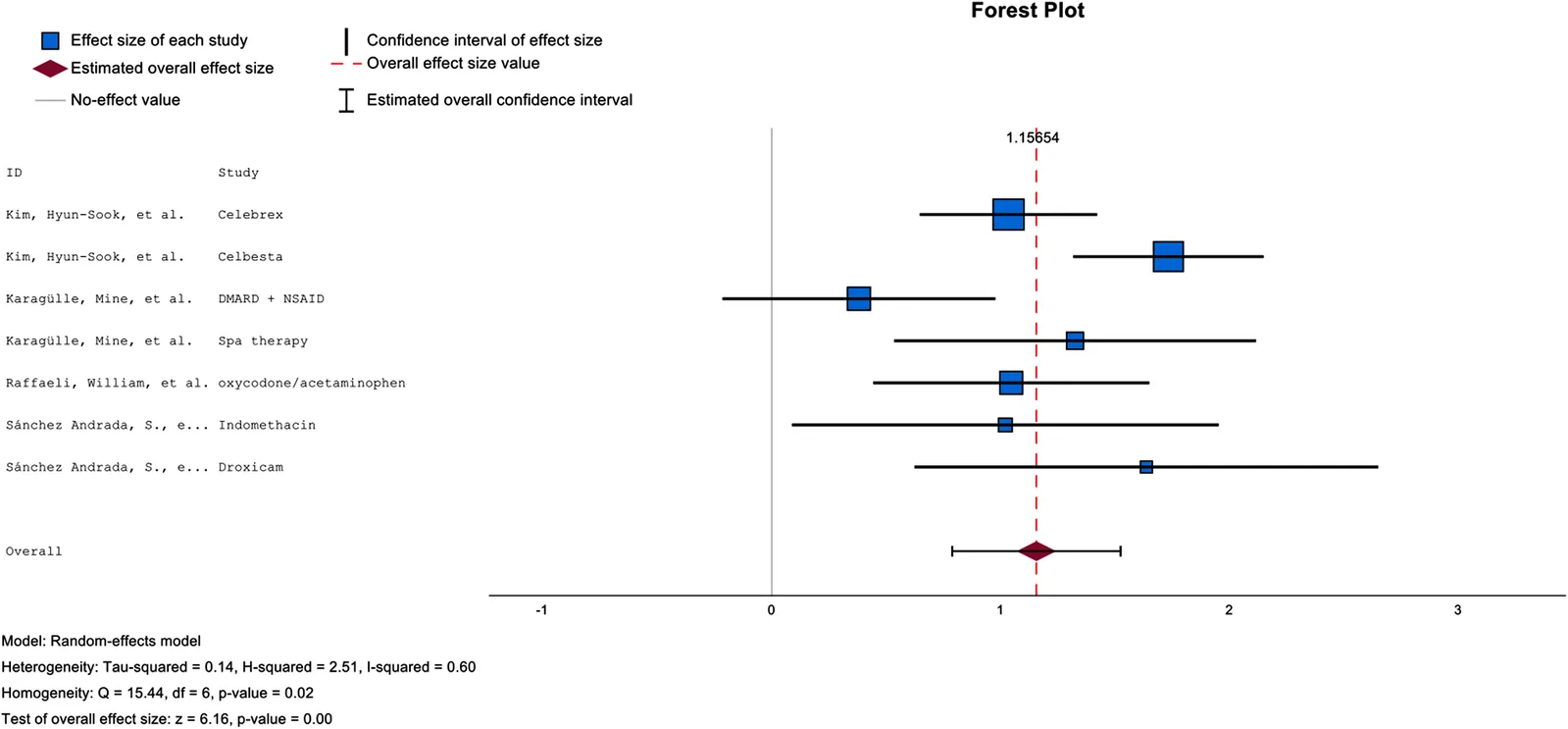
100-point pain scale meta-analysis results

Among the evaluated interventions, only DMARD + NSAID failed to show overall pain relief (*p* = 0.211). When comparing eligible treatment regimens to each other, celecoxib (Celbesta) demonstrated significantly greater pain relief (1.73, 95% CI: 1.317 to 2.149, *p* < 0.001). Meanwhile, while the remaining interventions (celecoxib (Celebrex), spa therapy, oxycodone/acetaminophen, indomethacin, and droxicam) improved pain scores, they did not show a significant advantage over other included therapies within the analysis (Fig. 8).

Significant heterogeneity was again observed in the 100-point group. The chi-square test indicated substantial overall variability (Q = 15.437, *p* = 0.017), with an I² of 60.2%, necessitating the use of a random-effects model. Results of the Egger’s regression test did not suggest significant asymmetry and potential publication bias (*p* = 0.133). While heterogeneity and publication bias improved in the 100-point group compared to the 10-point and 5-point group, likely due to an increased number of eligible data points, they remain potential limitations of the findings.

A systematic review of the eligible trials across three different pain scales yielded findings consistent with the meta-analysis. In the 5-point pain scale subgroup, etodolac (200 mg and 300 mg) and piroxicam demonstrated the greatest pain relief among all treatments. The efficacy of etodolac 200 mg twice daily was replicated across multiple trials [12, 14, 17] with varying but substantial success, reducing pain scores from baseline 35.29% − 77.78%. In contrast, ibuprofen 400 mg by mouth three times daily and ketoprofen 50 mg by mouth three times daily showed little to no improvement in pain scores [16].

For trials utilizing a 10-point pain scale, radial ESWT emerged as the most effective treatment, reducing pain scores by 61% from baseline [20]. Electroacupuncture also showed a high decrease in pain scores with 39.35% ^(18)^. Conversely, soft orthoses, semi-rigid orthoses shoe supports, and autogenic training provided little to no pain relief [18, 19]. Other medications, including naproxen, diclofenac, indomethacin, piroxicam, and milnacipran, demonstrated a < 15% pain score decrease from baseline after therapy [21, 22]. Results regarding piroxicam are especially of note due to substantially better pain relief being noted in an alternative study utilizing the 5-point pain scale.

In the 100-point pain scale subgroup, tocilizumab (TCZ) and Janus kinase inhibitor (JAKi) therapy resulted in the most substantial pain relief, with decreases of 65.6% and 80% in pain scores, respectively [28]. Other notable treatments included droxicam (65.1% decrease), nefopam (61% decrease), celecoxib (Celbesta) (55.9% decrease), and hyaluronic acid injection (53.1% decrease), indomethacin (48.53% decrease), spa therapy (47.15% decrease), etoricoxib 90 mg (43.92% decrease), celecoxib (Celebrex) (40.04% decrease), pelubiprofen (37.92% decrease), rituximab + MTX (36.47% decrease), celecoxib (33.13% decrease), etoricoxib 60 mg (33.11% decrease), oxycodone/acetaminophen (32.93% decrease), etoricoxib 30 mg (32.55% decrease), and ketoprofen (31.34% decrease). The remaining treatment options in this subgroup reduced pain scores by less than 30%, though all therapies contributed to overall pain reduction.

## Discussion

The findings from this systematic review and meta-analysis provide a comprehensive evaluation of analgesic therapies for rheumatoid arthritis (RA) related pain. When discussing results, it is essential to first establish the role of analgesics in the management of RA. As an autoimmune disorder with onset frequently occurring in younger adults, often earlier than other forms of arthritis, slowing disease progression is a primary therapeutic objective. DMARDs currently serve as first-line agents to achieve this goal in most patients. However, even in the context of well-controlled disease activity, many patients continue to experience chronic pain. Analgesics are therefore employed to address symptom control by reducing pain levels. Importantly, these agents do not influence the underlying disease process and should be used as adjunctive rather than primary therapy. This distinction is critical, as successful pain management is closely linked to the overall effectiveness of the underlying disease-modifying treatment strategy.

Against this clinical backdrop, the results of the meta-analysis identified differences in treatment regimens and their effectiveness in reducing overall pain scores. To account for variations in pain assessment methods, trials were analyzed based on their respective pain scales, categorizing them into 5-point, 10-point, and 100-point subgroups. Within the 5-point scale subgroup, etodolac and piroxicam provided significantly greater pain relief compared to acetylsalicylic acid and sodium salicylate. In the 10-point scale subgroup, ESWT was the only intervention that significantly reduced pain scores compared to alternative treatments. Lastly, within the 100-point scale subgroup, celecoxib (Celbesta) was the sole therapy that demonstrated a significant reduction in pain scores in comparisons to other treatments within this subgroup.

Findings from the systematic review, despite variations in pain scale methodologies, highlighted several key treatments as potentially effective options. Among pharmaceutical therapies, TCZ, JAKi, droxicam, nefopam, celecoxib (Celbesta), hyaluronic acid, etodolac, and piroxicam all demonstrated substantial pain reduction (≥ 50% decrease from baseline). However, etodolac and piroxicam were evaluated in multiple trials, and their efficacy was inconsistent across studies. Conversely, several commonly used pharmacologic agents, including naproxen, ibuprofen, diclofenac, DMARD + NSAID combinations, oxycodone/acetaminophen, and lidocaine, failed to achieve substantial pain reduction. While most of these treatments, except ibuprofen, demonstrated some degree of pain relief, their effectiveness was limited. Generally, NSAIDs are considered to be equally effective at appropriate doses in reducing pain, these results imply a potential underlying issue that could reside in a failure to optimally manage first line agents, such as DMARDs.

Among nonpharmaceutical interventions, ESWT demonstrated the most significant pain relief, followed by electroacupuncture. In contrast, soft and semi-rigid orthotic shoe supports and autogenic training provided little to no benefit in pain reduction.

Overall, the systematic review expanded the scope of analyzed treatment options while maintaining consistency with the meta-analysis findings. Across both approaches, etodolac, piroxicam, ESWT, and celecoxib (Celbesta) emerged as highly effective treatments. Additionally, the systematic review identified TCZ, JAKi, droxicam, nefopam, hyaluronic acid, and electroacupuncture as potential therapeutic options. However, these findings remain anecdotal, as they were not subjected to further statistical analysis.

These findings suggest that while traditional NSAIDs remain widely used in the symptomatic treatment of RA pain management, their clinical benefit is inconsistent among trials. The identified limited effectiveness of NSAIDs does raise concerns about their long-term utility, suggesting that their role should be carefully considered, particularly in patients with moderate-to-severe pain. However, while NSAIDs are generally considered comparable in their efficacy for RA pain management, potential limitations within the analysis and variability in study designs may have contributed to the inconsistent results. Certain agents, such as etodolac, piroxicam, celecoxib (Celbesta), and droxicam, did consistently demonstrate superior pain control compared to more commonly used NSAIDs such as ibuprofen, diclofenac, and naproxen. While the mechanism of action resulting in differing levels of efficacy is unknown, it is suggested that pain relief comes by way of cyclooxygenase (COX) enzyme inhibition, resulting in reduced inflammation and pain. These results, while potentially due to low evidence and limitations, emphasize the importance of NSAID selection when considering its use in an RA population.

Targeted therapies such as JAKi and TCZ are also recognized as effective treatment options for RA-related pain. JAKi reduces pain by modulating intracellular signaling pathways that are involved in inflammation. Meanwhile, TCZs effectiveness is through its antagonism of interleukin-6 (IL-6) receptors which leads the suppression of systemic inflammation. These alternative pathways to NSAID therapy demonstrated favorable results in our analysis and may represent reliable adjunctive options for patients with difficult-to-treat RA pain alongside first-line disease-modifying antirheumatic drugs (DMARDs).

Alternatively, in a patient population that wants to avoid typical pharmacological pain relief therapy, results suggest ESWT could provide pain relief as or more effective than management through medication. The mechanism of ESWT in pain relief is thought to involve the improvement of microcirculation, inhibition of inflammatory response, promotion of neural functional recovery, and stimulation of osteoblasts and chondrocyte activity. Electroacupuncture also provides patients and providers another non pharmaceutical method for moderate pain relief by way of nerve modulation resulting in the blocking of pain signals, induction of tissue repair, and decrease of inflammation. In contrast, therapeutic options such as autogenic training and orthotic shoe supports lacked the ability to address the inflammatory or neurological components of RA pain, limiting their overall effectiveness.

An important consideration in RA pain management is disease severity, treatment duration and prior medication use, which may include the effectiveness of a given therapy. While patients with early-stage RA may experience greater pain relief with NSAIDs, those with more moderate to severe disease progression may require alternative pain relief. This could explain why etodolac and piroxicam showed inconsistent results across trials, as their effectiveness may depend on the degree of the underlying condition. The duration of therapy is also critical, as some medications may require extended use to demonstrate their full analgesic effects. For instance, biologics such as TCZ and JAKi typically show optimal pain reduction over prolonged periods. Additionally, patients with a history of long-term NSAID use or opioid exposure may develop tolerance, leading to diminished effectiveness. This is a vital consideration when management for pain is long-term.

The findings of this study reinforce the importance of a personalized approach to RA pain management, where treatment selection should be based on disease stage, prior treatment history and patient-specific factors. Results also indicate opioids do not have to be a primary choice for pain relief, as many alternative therapies were able to show significant improvement in patients without the risk of opioid use disorder.

Advantages of this study include the use of a comprehensive search strategy that utilized multiple databases. Additionally, multiple trial designs, various pain scales and the inclusion of a diverse range of analgesic treatments allowed for a larger group of trials to be included and evaluated.

This analysis also contained several major limitations. High heterogeneity and publication bias was identified among all three pain scale subgroups. This likely occurred due to a combination of broad inclusion criteria and an overall lack of quality trials. The inclusion criteria, while allowing for the evaluation of many analgesic options, resulted in strong variability from dosing regimens, patient characteristics and overall study designs. Additionally, an already limited number of trials were then separated into three individually analyzed subgroups, further exacerbating heterogeneity and publication bias. Another limitation was the presence of potential bias within the trials. While most presented with a low risk of bias, five trials did display some concern while four trials had a high risk of bias. A final limitation of this analysis is the inconsistency in guideline-directed use of DMARDs across the included trials. The extent to which DMARD use was addressed varied substantially. Nine trials required participants to be on a stable dose of DMARDs for a defined period, typically between 8 and 12 weeks. Three trials permitted inclusion of participants taking DMARDs but did not require their use, resulting in mixed populations of both DMARD users and non-users. Thirteen trials did not specify DMARD use within their inclusion or exclusion criteria, nor was its use documented within the study populations. One trial explicitly excluded participants who had received DMARD therapy within the previous six months. Additionally, the use of various DMARDs was widely not specified, adding further to the lack of clarity between DMARD use and analgesic efficacy. This overall variability introduces a potential confounding factor, as analgesics are recommended in adjunct to first-line therapy, such as DMARDs. Furthermore, the use of DMARDs may influence pain outcomes by reducing disease activity and joint inflammation, leading to the potential overestimation of analgesic therapy related pain relief. The limited specificity of trials coupled with the complexity of the relationship between RA treatment and pain relief, limited the ability to adequately adjust for concomitant therapies. These limitations speak to the lack of consistent, high-quality trials currently available regarding pain in an RA population.

Nearly all risk of bias came way of a lack of blinding within the trial. These results suggest conclusions made from this meta-analysis may be unreliable and misleading. More research is needed to allow for a higher quality, comprehensive meta-analysis on the topic of analgesics in RA-related pain.

Future research should focus on standardizing pain assessment methods to reduce heterogeneity and improve comparability across studies, conducting high-quality randomized controlled trials in the RA population, and focusing on large-scale trials with long-term outcomes. Trials looking into treatments such as JAKi, TCZ, ESWT and multimodal approaches to optimize pain relief while minimizing side effects may be especially beneficial. In doing so, analgesic treatment strategies for RA-related pain could take on a more structured approach that emphasizes patient safety and long-term effectiveness.

## Conclusion

This systematic review and meta-analysis highlights key analgesic treatment options for RA-related pain. Overall, NSAIDs such as etodolac, piroxicam, celecoxib (Celbesta) and droxicam are associated with better pain relief than ibuprofen, naproxen and diclofenac. Meanwhile, targeted therapies such as JAKi and TCZ demonstrate strong efficacy, particularly for patients with moderate-to-severe RA. Extracorporeal shock wave therapy and electroacupuncture also showed promise as alternative, nonpharmaceutical treatments. However, limited high-quality trials resulted in potential trial bias, high heterogeneity and publication bias, which limits the reliability of these findings. Standardizing pain assessment methods and conducting large-scale, high-quality trials are essential for optimizing RA pain management and creating a standard of care in this patient population.

## Data Availability

All data generated or analyzed during this study are included in this published article. Additional data is available from the corresponding author on reasonable request.

## Abbreviations

RA: Rheumatoid arthritis
MDD: Major depressive disorder
DMARDs: Disease-modifying antirheumatic drugs
NSAIDs: Nonsteroidal anti-inflammatory drugs
RoB 2: Risk of Bias 2
ROBINS-I V2: Risk of Bias in Non-Randomized Studies, Version 2
CI: Confidence interval
VAS: Visual analog scale
ESWT: Extracorporeal shock wave therapy
TCZ: Tocilizumab
JAKi: Janus kinase inhibitor
MTX: Methotrexate
COX: Cyclooxygenase
IL-6: Interleukin-6
